# Enhancing performance in the presence of outliers with redescending M-estimators

**DOI:** 10.1038/s41598-024-64239-6

**Published:** 2024-06-12

**Authors:** Aamir Raza, Mashal Talib, Muhammad Noor-ul-Amin, Nevine Gunaime, Imed Boukhris, Muhammad Nabi

**Affiliations:** 1 Govt. College Women University Sialkot, Sialkot, Pakistan; 2 COMSATS University Islamabad-Lahore Campus, Lahore, Pakistan; 3 Department of Basic Sciences, College of Science and Theoretical Studies, Saudi Electronic University, 11673 Riyadh, Saudi Arabia; 4 Department of Physics, Faculty of Science, King Khalid University, P.O. Box 960, Abha, Saudi Arabia; 5 Khost Mechanics Institute, Khost, Afghanistan

**Keywords:** Outliers, Redescending M-estimator, Robust, Ordinary Least Square, Engineering, Mathematics and computing

## Abstract

In real-life situations, we have to analyze the data that contains the atypical observations, and the presence of outliers has adverse effects on the performance of ordinary least square estimates. In this situation, redescedning M-estimators, proposed by Huber (1964), are used to tackle the effects of outliers to increase the efficiency of least square estimates. In this study, we introduce a redescending M-estimator designed to generate robust estimates by mitigating the influence of outlier observations, even when the tuning constant is set to low values. This innovative estimator exhibits enhanced linearity at its core and maintains continuity throughout its range. Our proposed estimator stands out for its novelty, simplicity, differentiability, and practical applicability across real-world scenarios. The results of the proposed redescedning M-estimators are compared with existing robust estimators using an extensive simulation study. Two examples based on real-life data are also added to validate the performance of the suggested function. The formulated redescedning M-estimator produced efficient results as compared to all the considered redescedning M-estimators.

## Introduction

The ordinary least square (OLS) technique is usually applied to estimate the parameters of the regression line. The performance of the OLS depends on the assumptions of the error terms. The OLS procedure is widely applied in real-life situations for estimation, and it is regarded as an ideal technique for estimation. Nevertheless, it is recognized that OLS may perform less well when errors are not normally distributed, especially when heavy-tailed distributions or outlying data are included. The OLS’s sensitivity to outliers can produce deceptive results. The robust regression technique has been created as an improved alternative in response to these difficulties. Robust regression functions can be used as a supplement to the least squares estimate, address the impact of outliers and these methodologies can also identify valid observations and can provide stability in the presence of substantial data points. Robust regression becomes an essential technique when analyzing datasets impacted by outliers, as it allows the detection of such variations and produces consistent results when data have outliers.

Huber^1^ created M-estimation, the most widely used general robust regression approach. The robust estimators that are most commonly used are the following: Huber’s M-Estimators Huber^1^, MM-estimators (Yohai^2^), GM-Estimators, Siegel’s Repeated Median Estimators (Rousseeuw^3^), Least Median of Squares (LMS) estimators, Least Trimmed Squares (LTS) estimators (Rousseeuw^4^), S-Estimators (Rousseeuw^4^), Minimum Volume Ellipsoid (MVE) estimators (Rousseeuw^3^), and Minimum Covariance Determinant (MCD) estimators (Rousseeuw&Driessen^5^), robust estimators for exponential families of distribution (Baraud & Chen^6^) and robust estimators for high directional linear regression models (Ghosh et al.^7^).

## M-Estimators

The creation of the M-estimator embodies Huber’s fundamental contribution to statistical methods. Huber’s approach minimizes a function of residuals that develops more slowly, in contrast to traditional approaches that minimize the sum of the squared residuals. This method was created especially to lessen the impact of outliers in the regression analysis. The maximum likelihood formulations are used in M-estimators, which perform exceptionally well in non-normally distributed settings by figuring out the most optimal weightings for the dataset. To strengthen the estimator against the impact of outliers and improve the general resilience and reliability of regression analyses, this robust technique entails replacing the squared residuals used in Ordinary Least Squares (OLS) estimation with an alternative function of residuals, resulting in

1 $$\mathop {Minimize}\limits_{{\hat{\beta }}} \sum\limits_{k = 1}^{n} {\rho \left( {p_{k} } \right)}$$

where $\rho$ is a symmetric function with a unique minimum value at zero. The $\rho \left( {p_{k} } \right)$ is typically chosen to represent a weighting mechanism for the kth residual, deliberately giving outlier observations less weight. By purposefully reducing estimates, this intentional reduction makes estimates less vulnerable to noise, which strengthens the modeling method. Interestingly, giving a weight of zero to an observation correlates with classifying it as an outlier. Certain characteristics of a well-designed ρ function are necessary to ensure its reasonability within the strong regression framework.

1. $$\rho \left(0\right)=0$$
2. $$\rho \left(p\right)\ge 0$$
3. $$\rho \left(p\right)=(-p)(symmetry)$$
4. For $$0<{p}_{1}<{p}_{2}\Rightarrow \rho ({p}_{1})\le \rho ({p}_{2})$$
5. $\rho$ is continuous ( is differentiable)

By differentiating Eq. (1) with respect to the residuals *p*, resulting the psi function given as

2 $$\sum_{i=1}^{n}\Psi \left({p}_{i}\right){X}_{i}=0$$

where $\Psi \left(.\right)$ is the derivative of $\rho \left(.\right)$ and the maximum likelihood estimator serves as the corresponding M-estimator. After that, the weight function is obtained by dividing the psi function by the residuals that correspond to it, which is, ${w}_{i}=\frac{\Psi \left(p\right)}{{p}_{i}}$. Then the above estimated weight function can be written as

3 $$\sum_{i=1}^{n}w\left({p}_{i}\right){X}_{i}=0$$

Iterative methods are necessary to solve the above-described nonlinear equation system to compute M-estimators. When it comes to optimization tactics, the Iterative Reweighted Least Squares (IRLS) approach is widely acknowledged as a popular and widely used method. Because IRLS is iterative, it is a reliable and effective technique for handling the complexity involved in calculating M-estimators. The valuable contribution in the field of M-estimators is done by many researched and renowned names are Raza et al.^8^, Mukhtar et al.^9^, Luo et al.^10^, Anekwe & Onyeagu^11^, Noor-ul-Amin et al.^12^, Khalil et al.^13^, Alamgir et al.^14^, Ullah et al.^15^, Ali & Qadir^16^, Qadir^17^, Hampel^18^, Andrews^19^ and Beaton & Tukey^20^.

## Redescending M-estimators

One of the unique capabilities of redescending M-estimators is their complete ability to eliminate the impact of severe outliers. These estimators exhibit robustness when handling data with prominent outliers, as evidenced by their notable non-decreasing behaviour around the origin. The following list describes a few popular and highly known redescending M-estimators.

A first attempt at M-estimator specifically for regression tasks was presented by Andrews in 1974 by giving Andrews-Sine function that was a unique redescending M-estimator. This estimator is simply redescending and stands out for its increased robustness against outliers. The $\Psi$—function of Andrews- sine estimator is presented as 

4 $$\Psi \left( p \right) = \left\{ {\begin{array}{*{20}c} {hsign\left( {\frac{p}{h}} \right)} & {\left| p \right| \le h\pi } \\ 0 & {\left| p \right| > h\pi } \\ \end{array} } \right\}$$

where $p$ is the OLS error term and *h* is the tuning constant.

Beaton & Tukey^20^ introduced yet another redescending M-estimator known as Tukey’s bi-weight function, which has gained widespread utility. The Tukey’s bi-weight estimator’s $\Psi$—function can be obtained by

5 $$\Psi \left( p \right) = \left\{ {\begin{array}{*{20}c} {p\left[ {1 - \left( {\frac{p}{k}} \right)^{2} } \right]^{2} } & {\left| p \right| \le k} \\ 0 & {\left| p \right| > k} \\ \end{array} } \right\}$$

where *k* is tuning constant. Certain drawbacks of the Andrews Ψ function are efficiently addressed by Tukey’s bi-weight and. It’s crucial to remember that these substitutes provide some logical compliance a lesser weight.

Hampel^18^ is credited with introducing Hampel’s three-piece-wise redescending M-estimator, commonly referred to as Hampel’s three-part redescending estimators. These estimators are characterized by a psi-function that becomes 0 in the presence of significant residuals. Notably, the objective function of these M-estimators is a stepwise function, illustrating their efficacy in addressing outliers and efficiently handling regression problems. The $\Psi$- function of Hample’s M estimator is,

6 $$\Psi \left( p \right) = \left\{ {\begin{array}{*{20}c} p & {\left| p \right| \le k} \\ {ksign\left( p \right)} & {k < \left| p \right| \le h} \\ {k\frac{{n - \left| p \right|}}{{n - h}}sign\left( r \right)} & {h < \left| p \right| \le n} \\ 0 & {\left| p \right| > n} \\ \end{array} } \right\}$$

where *h*, *k* and *n* are tuning constants, 0 < *k* ≤ *h* < *n* < ∞. The Princeton Robustness Study revealed that his estimator performed admirably. The function is not optimal and it is not perfectly differentiable. One would prefer a smoother Ψ-function despite its success.

There has been a notable trend towards the development of techniques characterized by smoother mathematical properties and increased robustness. This trend has led to the creation of smoothly redescending M-estimators, which have been influenced by the preference for such characteristics. Qadir^17^ acquainted another redescending M-estimator comprehended as Qadir Beta function. The $\Psi$ funtion of Qadir Beta estimator is given by

7 $$\Psi \left( p \right) = \left\{ {\begin{array}{*{20}c} {\frac{p}{{16k^{4} }}\left( {k + p} \right)^{2} \left( {k - p} \right)^{2} } & {\left| p \right| \le k} \\ 0 & {\left| p \right| > k} \\ \end{array} } \right\}$$

According to research done by Ali & Qadir^16^, the author suggested a modified version of Tukey’s bi-weight function. This updated version’s $\Psi$—function is shown as.

8 $$\Psi \left( p \right) = \left\{ {\begin{array}{*{20}c} {\frac{{2p}}{3}\left[ {1 - \left( {\frac{p}{k}} \right)^{4} } \right]^{2} } & {\left| p \right| \le k} \\ 0 & {\left| p \right| > k} \\ \end{array} } \right\}$$

A redescending M-estimator was developed by Ullah et al.^15^ to identify outliers. The $\Psi$—function of Ullah’s redescending M- estimator is presented by

9 $$\Psi \left(p\right)= p{\left[1+{\left(\frac{p}{k}\right)}^{4}\right]}^{-2} for \left|p\right|\ge 0$$

Alamgir et al.^14^ indicated another redescending M- estimator for robust regression. The $\Psi$ -function of Alamgir’s redescending M- estimator is presented by

10 $$\Psi \left( p \right) = \left\{ {\begin{array}{*{20}c} {\frac{{16pe^{{ - 2\left( {\frac{p}{k}} \right)^{2} }} }}{{\left( {1 + e^{{ - \left( {\frac{p}{k}} \right)^{2} }} } \right)^{2} }}} & {\left| p \right| \le k} \\ 0 & {\left| p \right| > k} \\ \end{array} } \right\}$$

Khalil et al.^13^ also introduces a redescending M-estimator. The $\Psi$- fucntion of his estimator is presented by

11 $$\Psi \left( p \right) = \left\{ {\begin{array}{*{20}c} {p\left( {\frac{3}{2}} \right)\left\{ {1 - \left( {\frac{p}{k}} \right)^{4} } \right\}^{2} \sin \left[ {\left( {\frac{2}{3}} \right)\left\{ {1 - \left( {\frac{p}{k}} \right)^{4} } \right\}^{2} } \right]} & {\left| p \right| \le k} \\ 0 & {\left| p \right| > k} \\ \end{array} } \right\}$$

Noor-ul-Amin et al.^12^ proposed a new redescending M-estimator for robust regression whose $\Psi$- fucntion is given as

12 $$\Psi \left(p\right)=\frac{{c}^{2}}{4}\left[\frac{{tan}^{-1}{\left(\frac{2p}{c}\right)}^{2}}{4}+\frac{{p}^{2}{c}^{2}}{{c}^{4}+16{p}^{4}}\right]\, for \left|p\right|\ge 0$$

Another new redescending M- estimator was introduced by Raza et al.^8^, he claimed that his psi function provided more efficient results. The $\Psi$- function of Raza given below

13 $$\Psi \left(p\right)=\frac{{k}^{2}}{2a}\left[1-{\left\{1+{\left(\frac{p}{k}\right)}^{2}\right\}}^{-a}\right]\, for \left|p\right|\ge 0$$

For regression analysis, attaining the highest breakdown point is a desirable attribute of several redescending M-estimators. The M-estimator proposed by Ali et al. and Alamgir et al. are noteworthy due to its total rejection of observations with greater residuals. Although Ullah et al. attempt to offer an estimator that overcomes this constraint, it is not very generic. As a result, we have created an M-estimator with redescending characteristics that successfully fixes the issues with the estimators that were previously provided.

## Proposed redescending M-estimator

We present a novel redescending M-estimator with the characteristics of redescending estimators to improve the robust regression outlier identification. Specifically created to overcome the drawbacks of traditional M-estimators, this new estimator is a cutting-edge tool for robust regression. The following explanation delves further into the characteristics and forms of the relevant $\Psi$- function, weight function, and $\rho$ -function, offering a clear understanding of the unique aspects of our suggested methodology.

**Figure 1:**
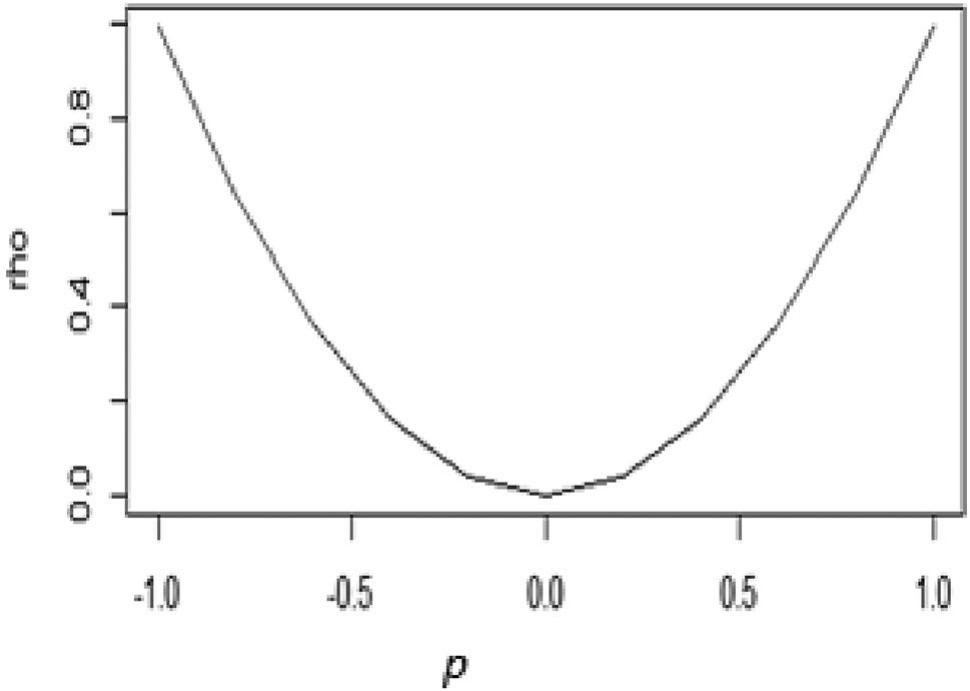
Graph of the proposed objective function.

The proposed M-estimator’s function is specified as

14 $$\rho \left( p \right) = \left\{ {\begin{array}{*{20}c} {\frac{{p^{2} }}{{810a^{8} }}\left[ {p^{8} - 30a^{2} p^{4} + 405a^{2} } \right]} & {if\left| p \right| \le a} \\ {\frac{{188a^{2} }}{{405}}} & {if\left| p \right| > a} \\ \end{array} } \right\}$$

where $p$ are residuals obtained from the OLS *a* works the tuning constant. We have discussed in details the shape of the objective function in Fig. 1. The suggested function is recursive and meets the required criteria. The typical characteristics are:

- $$\rho \left(0\right)=0$$
- $$\rho \left(p\right)\ge 0$$
- $$\rho \left({p}_{i}\right)=\rho (-{p}_{i})(symmetry)$$
- For $$0<{p}_{i}<p\Rightarrow \rho ({p}_{i})\le \rho ({p}_{j})$$
- $\rho$ is continuous ( is differentiable)

A series of residuals are produced using the R-program to show how well the suggested M-estimator performs. The objective function’s resulting graph is shown in Fig. 1, and it clearly shows a declining trend. This graphic sheds light on the properties and performance of the suggested objective function.

Differentiating $\rho \left( . \right)$ with respect to residuals, we obtained psi-function that is represented in Eq. (15)

15 $$\Psi \left( p \right) = \left\{ {\begin{array}{*{20}c} {p\left[ {1 - \left( {\frac{p}{{\sqrt 3 a}}} \right)^{4} } \right]^{2} } & {if\left| p \right| \le a} \\ 0 & {if\left| p \right| > a} \\ \end{array} } \right\}$$

**Figure 2:**
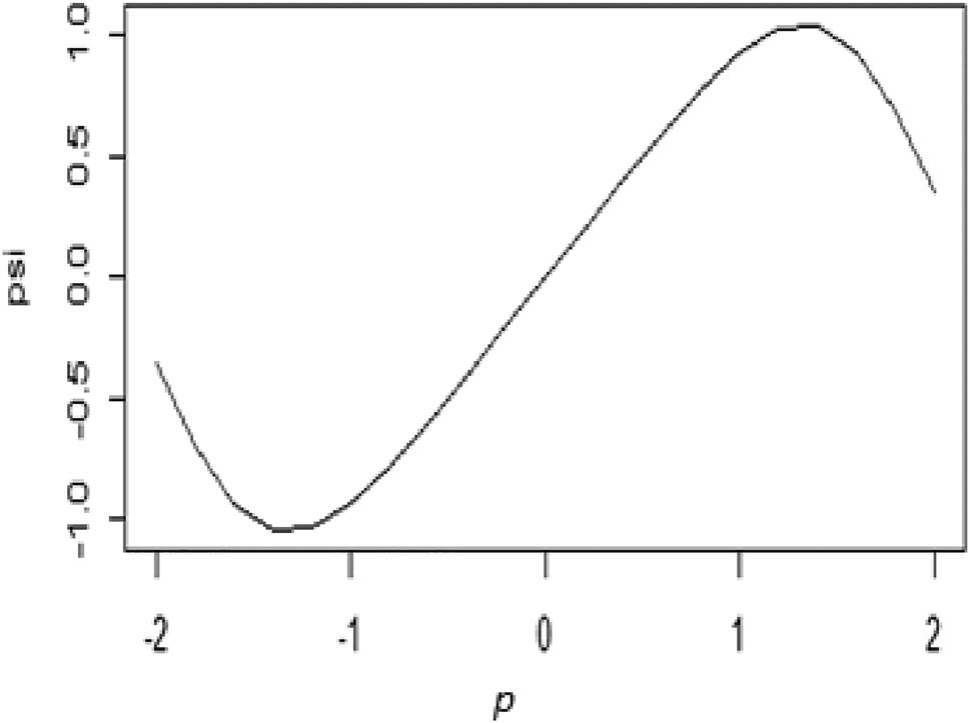
Graph of Ψ—function for the suggested M-Estimator.

Using data in Fig. 1, the graphical display of $\Psi \left(p\right)$ is presented in Fig. 2.

The recommended psi-function given in Fig. 2 is a more linear and differentiable profile at the center of the curve than the considered psi-functions used for robust regression to tackle the outliers. Crucially, the proposed psi-function fulfills all the necessary conditions to build an iterative M-estimator. Additionally, this function highlights how well it captures the desired qualities for robust regression analysis by giving greater weight to values closer to the center and decreasing weight for values away from the center.

We found the corresponding weight function, which is provided, by dividing the $\Psi$-function by residual "r".

16 $$w\left( p \right) = \left\{ {\begin{array}{*{20}c} {\left[ {1 - \left( {\frac{p}{{\sqrt 3 a}}} \right)^{4} } \right]^{2} } & {if\left| p \right| \le a} \\ 0 & {if\left| p \right| > a} \\ \end{array} } \right\}$$

**Figure 3:**
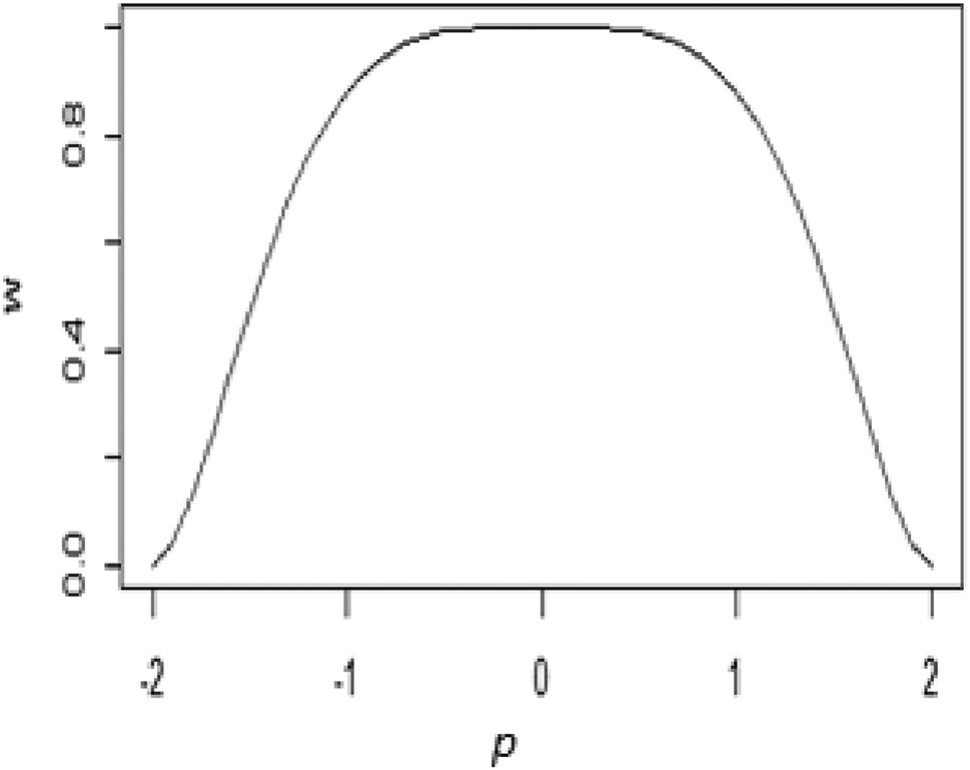
Graph of the weight -function for Proposed M-estimator.

The graphical representation of the weight function is displayed in Fig. 3.

Figure 3 demonstrates the proposed weight function’s special robustness attributes.

## Graphical comparison of proposed redescending M-estimator

**Figure 4:**
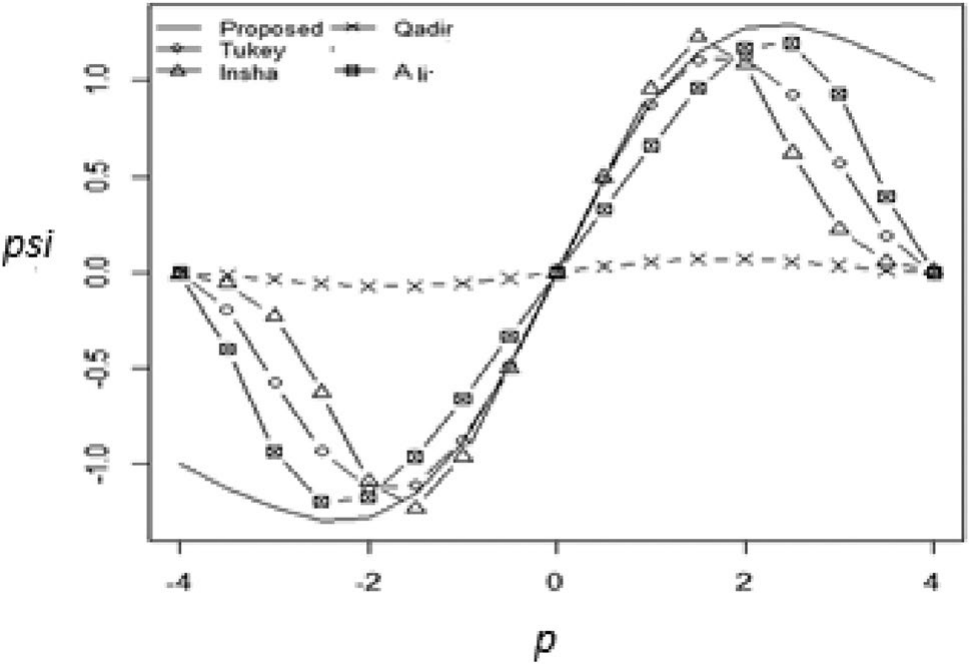
Combined plot of Tukey, Qadir, Ali, Ullah and Proposed Ψ –functions.

A comparative study is carried out between the suggested redescending M-estimator and other well-known redescending $\Psi$-functions using graphical representations of $\Psi$-functions. Especially, the suggested M-function showed linearity greater than the considered regressive functions, guaranteeing continuous differentiability everywhere. This property increases the suggested M-estimator’s overall efficacy. The graphical comparison between the proposed and existing $\Psi$-functions is clearly illustrated in Fig. 4, which further clarifies the beneficial aspects of the recommended methodology.

The Fig. 4 representations showed that the suggested psi function gave more weight to the central observations as compared to the considered estimators and less weight to the values that have larger outliers which is the primary objective of the proposed redescending M-estimator. The suggested estimator is continuous and differentiable everywhere within the graph.

## Practical applications

In this section performance of the suggested M-estimator is demonstrated by adding detailed comparisons among the considered redescending M-estimators by providing a thorough study of real-life data examples along with simulation results. By evaluating real-world data and simulating situations, our goal is to offer a thorough grasp of the effectiveness and relative benefits of the suggested estimator in real-world applications. To attain this objective R-programing is used.

### Example-1: yearly average price growth in China 1940–1948

In case I, the data is taken from the Rousseeuw’s^3^. This data had previously been employed by Ullah et al.^15^ and Raza et al.^8^ in their studies on robust regression. The dataset includes nine average annual rates of price growth that were observed between nineteen forty to nineteen forty eight. The variable of interest is the annual growth rate, and years are taken as the predictors. The average growth rates were 1.620%, 1.630%, 1.900%, 2.640%, 2.050%, 2.130%, 1.940%, 15.500%, and 364.0%.

Additionally, the war, the budget deficit, and increased government expenditure during that time all contributed to an exponential rise in prices that resulted in an important increase in rate jump to 364.00% in 1948..The suggested methodology is compared to other well-known robust methods using the cited data. Table 1 provides the estimates of sum of squared of errors and regression coefficients for every method. A thorough examination of the outcomes shows that OLS has serious performance issues and consistently showed adverse results. This emphasizes how sensitive OLS is towards the outliers, as it shown by the highest sum of squared errors (78532.88).

**Table1:** Comparison Among the existing and Proposed M-Estimators using data of Annual Average growth rate of China.

Methods	Coefficients estimates			
	*A*	*b*	Values used	*SSE*
OLS	− 1049	24.850	9	78,532.88
LMS	− 2.470	0.1020	7	0.69534
Tukey(2)	− 2.7535	0.10896	7	0.616652
Andrews(0.58)	− 2.7542	0.10898	7	0.616687
Ali (3.0)	− 2.7792	0.10956	7	0.617851
Qadir (1.0)	− 2.7779	0.10950	7	0.617785
Khalil (2.0)	− 2.7851	0.10970	7	0.618139
Alamgir (3.0)	2.7853	0.10970	7	0.61815
Ullah (1.5)	− 2.7851	0.10970	7	0.615962
Raza(8, 2)	− 2.6486	0.10656	7	0.611112
Proposed(1)	− 2.7832	0.10965	7	0.611111

On the other hand, other strong methods successfully lessen the influence of irregularities. Interestingly, the suggested robust function performs better on outlier-filled data, as evidenced by the lowest sum of square residuals. This emphasizes how reliable and effective the suggested estimator is as compared to other alternative choices.

### Example-2: data of telephone phone calls from Belgium from 1950–1973

The second case study, which comes from Rousseeuw’s^3^ article, looks at the Belgium Statistical Survey’s year-by-year count of international telephone calls (in 10 millions) made from Belgium between 1950 and 1973 (Table 2). There are few outliers in the response variable in this data. The years are the independent variable (represented by X), and the dependent variable (represented by Y) is the annual telephone call count. Several writers have previously used this dataset in their research: Qadir^17^, Ali & Qadir^16^, Khalil et al.^13^, and Raza et al.^8^. Table 2 presents a study of the performance of the suggested redescending M-estimator, taking into account estimators that are relevant to this dataset.

**Table 2:** Number of telephone calls (10 million) from the Belgium.

Years	1950	1951	1952	1953	1954	1955	1956	1957
Calls	0.440	0.470	0.470	0.590	0.660	0.730	0.810	0.880
Years	1958	1959	1960	1961	1962	1963	1964	1965
Calls	1.060	1.200	1.350	1.490	1.610	2.120	11.90	12.40
Years	1966	1967	1968	1969	1970	1971	1972	1973
Calls	14.20	15.90	18.20	21.20	4.300	2.400	2.700	2.900

Table 3 presents a comprehensive comparison between our proposed robust estimator and Ordinary Least Squares (OLS) alongside other robust estimators. The regression coefficients were derived by applying all considered functions, with the corresponding sum of squares displayed in the table. Notably, outlier influence significantly compromises OLS estimates, resulting in the highest total of squared errors (659.44) and potentially misleading insights across the dataset. Conversely, outlier effects are effectively mitigated by all other M-estimators, including the robust estimator we have introduced. It is important to highlight that our proposed estimator exhibits the lowest sum of squared errors, indicating superior performance and yielding a model with minimized SSE. This underscores the efficacy of the recommended robust estimator in enhancing the accuracy and reliability of the regression model.

**Table 3:** Comparison among the existing and proposed M-estimators on the data of telephone calls.

Method	Coefficients			
	*a*	*b*	*n*	*SSEs*
LMS	− 5.1640	0.1080	16	0.13130
Tukey (3.8)	− 5.2439	0.1102	16	0.13665
Andrews (1.5)	− 4.9072	0.1033	16	0.17308
Ali (3.0)	− 5.2092	0.1094	16	0.13404
Qadir (1.0)	− 5.2092	0.1122	16	0.13404
Ullah (4)	− 5.2430	0.1102	16	0.14060
Khalil (4.0)	− 5.2343	0.1099	16	0.13863
Alamgir (3.0)	− 5.2343	0.1102	16	0.14127
Raza(6, 3.5)	− 5.5109	0.1094	16	0.13256
Proposed(2)	− 5.16568	0.1085	16	0.13120

**Figure 5:**
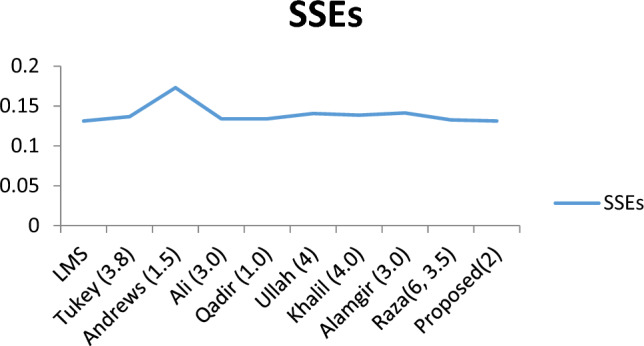
Graphical Comparison among the Existing and Proposed M-Estimators.

The Fig. 5, showed a graphical comparison of all the consider robust estimators along with proposed robust estimator. It showed that the proposed robust estimator has least sum of square of errors among all the considered robust estimators hence it produced the most efficient results for the data having contamination of outliers.

## Simulation results

The evaluation of performance among the considered estimators is frequently conducted through simulation, a valuable technique, particularly when the true parameter values of the generated data are known. Utilizing a simulation strategy devised by Rousseeuw^3^, we have assessed the effectiveness of the proposed method. We contrasted it with several prominent redescending M-estimators to gauge its performance against established benchmarks. For the simulation study, following OLS model is incorporated to generate the data

$$y_{i} = \alpha + \beta x_{i} + u_{i}$$

where *x* follows a normal distribution with $\mu = 20\,\& \sigma^{2} = 10$, $\alpha = 2$,$\beta = 1$ and $u_{i} \sim N\left( {0,1} \right)$. With the help of the previously mentioned linear model, we have produced a population of 10,000 values. The R-Program has then been used to randomly select 100 values from this population as a sample. The parameter estimates have been computed using the considered M-estimators in addition to the suggested redescending M-estimator. Table 4 presents the results, providing a summary of the parameter estimates derived from the various estimators that may be compared. In this study, we assess the performance of the specified functions under two distinct scenarios. Firstly, parameter estimation is carried out using clean, outlier-free normal data. Secondly, estimation is performed with the addition of 10% outliers in the Y direction. As discussed by Norazan^21^, M-estimators often yield suboptimal estimates in the presence of outliers in the X direction. The results presented in Table 4 represent the average outcomes of 50,000 simulated data iterations, each comprising 100 samples. All methodologies demonstrate consistent performance in Case 1, where outliers are absent, underscoring their reliability in standard settings. In contrast, our proposed M-estimator yields estimates closely resembling the actual parameter values utilized in the simulation for Case II, where outliers are intentionally introduced. With the exception of the OLS approach, which exhibits unreliable outcomes in the presence of outliers, the considered estimators consistently deliver efficient results, corroborating the findings outlined in Section. “Graphical Comparison of Proposed Redescending M-Estimator”. The demonstrated effectiveness of the suggested M-estimator, particularly in scenarios with limited sample sizes, underscores its reliability and robustness for sampling and estimation tasks.

**Table 4:** Comparison among the existing and proposed M-estimators for n = 100.

Method	*Case-I*		*Case-II*	
	*Const*	*X*	*Const*	*X*
OLS	1.99842	1.00002	7.04260	0.99794
Tukey (4.69)	1.99742	1.00011	1.99586	1.00018
Hample (4)	1.99742	1.00008	1.99586	1.00045
Huber (4)	1.99834	1.00002	1.99760	1.00023
Andrew(3.2)	1.99801	1.00021	2.19465	1.00004
Ullah (4)	2.00924	0.99964	1.99743	0.99764
Ali (4)	2.00303	0.99984	1.99923	1.00011
Khalil (5)	2.02813	0.99846	2.00358	0.999761
Raza (2)	1.99736	1.00009	1.99331	1.00047
Proposed	2.004547	0.99971	2.00341	0.999912

## Conclusion

The main purposed of this work is to obtain reliable and efficient estimates when data contain outliers. It has been consistently shown by the results from earlier sections that the proposed redescending M-estimator is more adaptable and effective than the redescending M-estimators considered in this study. The proposed M-estimator exhibits much more continuous behavior before redescending as compared to the previously developed redescending M-estimators. Greater breadth, flexibility, simplicity, and faster convergence characterize the proposed estimator above its predecessors. The suggested redescending M-estimator is more successful, producing the minimum sum of squared errors in the presence of outliers, according to real data applications. The results of simulation tests demonstrate that the coefficients produced by the suggested robust estimator are in good agreement with the genuine parameters, demonstrating the robust estimator’s comparability with other well-known estimators including Huber, Hampel, Andrews, and Beaton & Tukey. Furthermore, the suggested estimator performs no less well than the Ordinary Least Squares (OLS) method when there are no outliers. Together, these findings highlight the robustness and superiority of the proposed redescending M-estimator in a variety of scenarios and applications. The proposed redescending M-estimators can be utilized for combined monitoring the population mean and variance in simple random sampling, in two phase sampling, in ranked set sampling etc. This M-estimator can also be used to produce robust statistical quality control tools for SPC.

## Data Availability

The datasets used and/or analyzed during the current study are available from the corresponding author upon reasonable request.
